# A framework for assessing global health impacts of polar change: An urgent call for interdisciplinary research

**DOI:** 10.1007/s13280-025-02255-0

**Published:** 2025-11-07

**Authors:** Netra Naik, Karol Bot, Gail Whiteman, Lora E. Fleming, Karyn Morrissey, Richard Garth James Bellerby, Sam Dupont, Dmitry Yumashev, Susana Hancock, Brendan M. Rogers, Kristie L. Ebi, Joacim Rocklöv

**Affiliations:** 1 Arctic Basecamp Foundation, Rotterdam, The Netherlands; 2 University of Exeter Business School, Exeter, UK; 3 European Centre for Environment and Human Health in the University of Exeter Medical School, Penryn, Cornwall UK; 4 Discipline of Economics, J.E Cairnes School of Business and Economics, University of Galway, Galway, Ireland; 5 Norwegian Institute for Water Research, Bergen, Norway; 6 Faculty of Applied Sciences, UCSI University, Kuala Lumpur, Malaysia; 7 University of Gothenburg, Göteborg, Sweden; 8 Small World Consulting, Lancaster, UK; 9 Woodwell Climate Research Centre, 149 Woods Hole Road, Falmouth, MA 02540 USA; 10 University of Washington, Center for Health and the Global Environment, 3980 15th Avenue NE, Seattle, WA 98195 USA; 11 Heidelberg Institute of Global Health & Interdisciplinary Centre for Scientific Computing, Heidelberg University, Heidelberg, Germany

**Keywords:** Climate tipping points, Global health risks, Health frameworks, Polar physical changes, Regional health risks

## Abstract

Research on the human health risks of climate change is expanding, yet the influence of polar region shifts on these risks remains underexplored. This paper presents a framework to assess global and regional health risks stemming from polar physical changes. The polar regions are experiencing rapid environmental transformations, including melting ice, ocean warming, ocean acidification, permafrost thaw, intensifying wildfires, and alterations to jet streams, ocean currents. These changes can amplify global risks, affecting human health even in distant regions. The paper identifies potential cascading impacts on health and well-being via drivers such as extreme weather, heat stress, air, water quality, food supply, safety, vector ecology, and sea-level rise. A scoping review was conducted by an international team of public health and polar experts to support thematic categorization of regional and global health risks. The paper advocates integrating these amplified risks into health impact assessments through interdisciplinary, international collaboration to inform future policy responses.

**Supplementary Information:**

The online version contains supplementary material available at 10.1007/s13280-025-02255-0.

## Introduction

Research on the risks of climate change on human health is growing alongside increasing evidence of impacts (Rocque et al. 2021). Nearly 25% of global deaths are associated with environmental factors, including air pollution, non-potable water and extreme weather events. The World Health Organization (WHO) estimates that between 2030 and 2050, climate change directly or indirectly caused by human activities (e.g. burning fossil fuels, deforestation) could cause approximately 250,000 additional deaths per year, from malnutrition, malaria, diarrhea and heat stress alone (World Health Organization 2019).

Direct health effects of climate change result from rising temperatures, heatwaves, sea-levels and increases in the frequency of complex extreme weather events (such as windstorms, floods, droughts, and wildfires); and include for example, mortality from these extreme temperature events (hot and cold), and physical injuries because of extreme weather events (Watts et al. 2015; Cisse et al. 2022). Additionally, along with the direct effects, the indirect effects of climate change can impact health and well-being because of alterations in environmental conditions, for example through the spread of disease vectors, food insecurity and under-nutrition, displacement, and mental health (Watts et al. 2015; Cisse et al. 2022).

An underexplored area is how the drivers of health threats arise directly and indirectly from physical climate feedbacks from the Arctic and Antarctic, which have major functions governing the dynamics of the global climate system and are warming disproportionately faster than the rest of the Earth (Rosser et al. 2024). Further, the polar regions may experience potential climate tipping elements related to crucial thresholds in Earth’s climate system (Armstrong McKay et al. 2022; Lenton et al. 2023; Rosser et al. 2024). Several of these are likely to be triggered before reaching + 2 °C of global warming, resulting in non-linear future amplification of warming, regional cooling, shifts in precipitation patterns, or sea level rise. (Armstrong McKay et al. 2022). These include the irreversible melting of the Greenland and West Antarctic ice sheets, thawing of Arctic permafrost, abrupt loss of Barents Sea ice, and the collapse of the Labrador Sea subpolar gyre (Armstrong McKay et al. 2022; Lenton et al. 2023; Rosser et al. 2024).

This paper highlights the critical importance of the changing polar regions and examines how rapid and widescale changes in the Arctic and Antarctic can directly and indirectly impact human health and well-being regionally and globally. To this end, we first classify the human health risks of physical polar change into three categories: Regional direct and indirect health risks, global direct health risks, and global indirect health risks. Based on these classifications, the review develops a novel conceptual framework to better understand the relationship between changing polar regions and regional and global health risks.

## Methods

The methods included two stages: 2.1 Scoping Review and 2.2 Thematic Categorization and Framework Development.

### Scoping review

For this Study, the definition and methodology of a scoping review was informed by Mak and Thomas (2022), who describe it as a form of knowledge synthesis that iteratively maps and integrates existing or emerging literature on a particular subject. Scoping reviews are typically undertaken to help inform research questions in emerging or under researched areas or as a precursor to a more in-depth review, such as a systematic review. As will be outlined below in more detail, the scoping review was carried out in four stages that involved: (1) identifying the research question, (2) identifying relevant studies, (3) study selection, and (4) charting the data.

#### Identifying the research question

Emerging from a series of discussions on the links between climate change related changes in polar environment and potential human health impacts, the research question was developed collaboratively by an interdisciplinary advisory board of experts in public health, climate science, and polar change. The final research question reflected this objective:

*“Can human health risks be linked to physical polar changes (PPC), and if so, what are the manifestations of these impacts now and in the future?”*

Based on the identification of the final research objective, and the relative paucity of papers addressing this research question, a scoping review rather than a systematic review was identified as an appropriate method to capture the current evidence emerging in this area.

#### Identifying relevant studies/screening process

Electronic databases (Web of Science, PubMed, Scopus, Consensus, Science Direct, Nature) were searched to capture interdisciplinary studies from the last 15 years (2009–2025), covering climate science, public health, indigenous knowledge, and socio-economic factors. Keywords related to polar changes (e.g. Arctic ice melt), health risks (e.g. vector-borne diseases, mental health), and global climate change consequences (e.g. sea-level rise) were used (Table S1). To ensure that the keywords were comprehensive in capturing the breadth of studies involving polar change, climate change and health risks, search terms were based on recommendations from the co-authors consisting of an international team of health and climate scientists. The specific keywords (search strings) and filters applied per database are listed in the (Table S2).

The initial pool of articles retrieved through the database search were downloaded in CSV format and uploaded to Rayyan^1^, a free AI assisted review platform (Ouzzani et al. 2016). Three research team members (NN, KB, SH) conducted the initial title and abstract screening to assess relevance based on predefined keywords, inclusion criteria, and alignment with the research question. Throughout this stage, Rayyan was utilized to support the screening process, facilitating efficient organization, duplicate detection, tagging, and decision tracking. After removing duplicates and filtering out irrelevant studies, a curated list of potentially relevant articles was created. The list was subsequently circulated among all co-authors for consensus, after which three research team members (NN, KB, SH) conducted a full-text screening using Rayyan to confirm the final selection of studies (see flowchart in Fig. S1).

#### Study selection

Articles meeting the inclusion criteria were selected to categorize health risks and link them to polar physical changes (Table S3). Decisions to exclude were reviewed by at least two members of the research team. Only English language peer-reviewed scientific articles, government and industry papers, fact sheets from recognized climate and/or environmental organizations like World Health Organization were included.

To ensure comprehensive coverage, additional journals and resources were manually searched and included based on recommendations from subject matter experts in polar science and global health, enabling the inclusion of relevant studies that were not captured through standard academic database searches. This step is consistent with established scoping review frameworks (Arksey and O’Malley 2005; Levac et al. 2010) and was deemed necessary due to the paucity of peer-reviewed literature specifically linking polar change and global health. All included articles were peer-reviewed by the research team to accurately represent the academic landscape. Eligible articles included primary research utilizing quantitative, qualitative, and/or mixed methods approaches.

#### Charting the data

The research team members (NN, KB, SH) extracted the following data from the included articles: the health risk category (direct, indirect, regional and global), the physical environmental driver associated with the health risk and its link to polar physical changes. Data extraction for each article was performed by a minimum of two research team members (pair of health and climate expert); with any disagreements resolved through consultation with an additional team member to reach a consensus.

### Thematic categorization and framework development

**Fig. 1:**
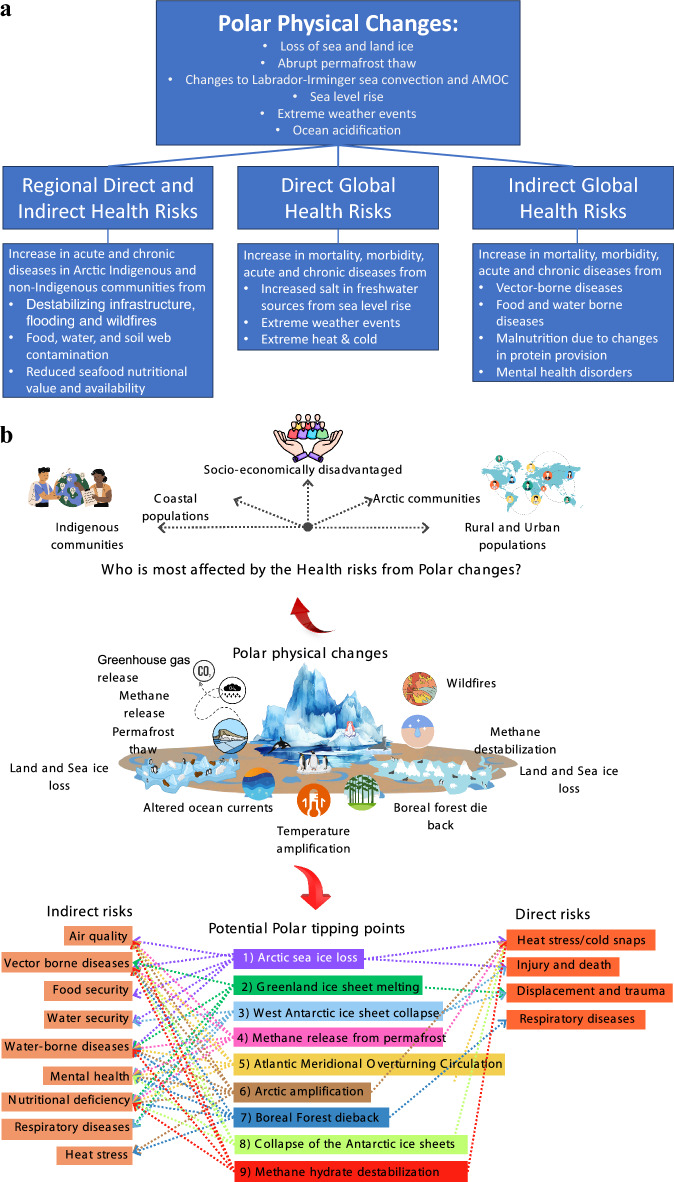
**a** Conceptual framework: showing the linkages between polar physical changes and direct and indirect, regional and global health risks. The examples included in the figure are not exhaustive. To note that chronic diseases, respiratory included, are affected via both direct exposure (heat/cold, smoke, pollution) and indirect systemic effects (nutrition and infection dynamics). **b** Direct and indirect health risks from polar tipping points. While polar changes affect global populations, those most at risk are communities with overlapping vulnerabilities, such as Arctic, coastal, Indigenous, rural, urban, and socio-economically disadvantaged groups due to greater exposure, limited resources, and factors like age, health, ethnicity, and education

Health risks were categorized into regional, global direct and indirect pathways following the definition provided by Watts et al. (2015). A conceptual framework was developed, expanding on the World Health Organization’s (WHO) Driver, Pressure, State, Exposure, Effect, and Action (DPSEEA) framework to address polar-specific gaps. The framework (Fig. 1a) for assessing the health risks of physical polar change classifies health risks into three broad categories.

- **Regional Direct and Indirect Health Risks encompass** human health risks to Arctic Indigenous and non-Indigenous communities, including but not limited to exposure to harmful contaminants released into water, soil, and food chains due to flooding, ocean acidification and permafrost thaw (see Sect. “Regional direct and indirect health risks in the Arctic”).
- **Direct Global Health Risks focus** on risks posed by sea level rise and changes to global weather patterns driven by polar physical changes. The sub-categories discussed are sea level rise and health, extreme weather, extreme heat and extreme cold (see Sect. “Direct global health risks”).
- **Indirect Global Health Risks** address risks resulting from altered environmental conditions caused by polar physical changes. The sub-categories discussed are vector-borne diseases, food and water borne diseases, and mental health (see Sect. “Indirect global health risks”).

The sub-categories listed above in the direct and indirect regional and global health risks are non-exhaustive.

## Results

The results from the scoping review were used to inform the conceptual framework describing the polar physical changes and the corresponding direct and indirect, regional and global, health risks is presented below (Fig. 1a, Fig. S1, Table S4).

Of the 282 articles identified through the database search, 195 were excluded. The articles were excluded based on predefined criteria (see Table S2), including non-English publications, literature published before 2009, and studies not addressing health impacts related to climate or polar change, or those with an overly narrow demographic focus. Out of 89 articles that met the inclusion criteria, 22 addressed regional health risks (24.71%), 44 focused on direct global health risks (49.43%), and 23 examined indirect global health risks (25.84%). Furthermore, of the 89 studies identified, 5 (5.6%) incorporated traditional or local knowledge, and this number remained unchanged following AI-assisted and manual screening, highlighting the limited integration of such perspectives.

### Polar physical changes and feedback loops

The Arctic is warming at an average rate of up to four times faster than the global average (Rantanen et al. 2022), a phenomenon known as “Arctic Amplification”, that is resulting in significant sea ice loss and rising greenhouse gas emissions from thawing permafrost and wildfires (Hugelius et al. 2024; See et al. 2024; Virkkala et al. 2025). The Antarctic is warming at an average rate of two times faster than the global average (Casado et al. 2023).

The warming induced decline of Arctic Sea ice decreases land and ocean albedo from shrinking snow, expanding vegetation, and melting glaciers and may prevent future ice ages (Meier and Stroeve 2022). This allows for greater solar radiation absorption and radiative warming, which intensifies global warming. Projections indicate that an ice-free Arctic could shift the timeline for crossing critical warming thresholds (2 °C above pre-industrial levels) earlier by nearly 25 years, while also accelerating permafrost thaw (Pistone et al. 2019; Thackeray and Hall 2019). Recent research shows a 62% chance of triggering multiple tipping points, some within the next decade, under current SSP2-4.5 policies, including Arctic summer sea-ice loss, Greenland ice sheet melt, and abrupt permafrost thaw (Deutloff et al. 2025).

Permafrost thaw, which is not fully factored into global carbon budgets and Earth system models (Turetsky et al. 2019), could consume as much as 10–20% of the remaining emissions allowance (carbon budget) to stay below + 2 °C (Treharne et al. 2024) when accounting for ‘gradual’ permafrost thaw, abrupt thaw events such as thermokarst, and intensifying wildfire regimes, further accelerating climate change. As permafrost thaws, it releases pollutants (e.g. methylmercury, Polychlorinated Biphenyls [PCBs]) (Yang et al. 2016), industrial chemicals and greenhouse gases (carbon dioxide and methane), creating a self-reinforcing loop of warming and further permafrost thaw (Schuur et al. 2022). Thawing permafrost further releases microorganisms (including viruses) and nuclear waste that contaminate soil, water and food webs (Miner et al. 2021), while rising temperatures facilitate vector-borne diseases spreading to new regions (Pavia et al. 2024).

Arctic surface temperatures are currently increasing at an average rate of + 0.55 to + 0.66 °C per decade, compared to the global average of + 0.19 °C (Li et al. 2023). This warming has led to unprecedented events such as rainfall at Summit Station in Greenland (National Snow and Ice Data Centre 2021), extreme temperature variations in Alaska, and systemic ecosystem changes in the Barents region and the northern Bering Sea (Overland et al. 2021). The reduction of summer Arctic Sea ice continues at an alarming rate (Cai et al. 2021), with a nearly ice-free Arctic in September projected as early as 2049 under the SSP2-4.5 scenario and 2043 under the higher-emission SSP5-8.5 scenario (Zhou et al. 2022). Loss of Arctic snow and ice could increase global warming by up to 44% (Marcianesi et al. 2020). Furthermore, the complete melting of the Greenland Ice Sheet could occur within a century and contribute an additional 7 m to global sea level rise (National Snow and Ice Data Centre 2021). At least 27 cm of sea level rise is now locked in due to current CO_2_ levels (Box et al. 2022).

At the same time, the Antarctic is also experiencing significant warming, with mean temperatures rising over + 3 °C from 1951 to 2018; this is three times the global average increase of 1 °C during the same period (Hughes et al. 2021). This warming has led to glacier retreat, ice shelf collapses, and increasing precipitation falling as rain rather than snow, as well as greening along the Antarctic Peninsula and Scotia Arc between the Antarctic Peninsula and South America (Hughes et al. 2021).

Antarctic sea ice shows more variability, due to factors such as atmospheric circulation patterns, oceanic processes, and regional differences, with less multiyear ice compared to the Arctic Ocean (Yu et al. 2021). Since November 2016, the decline in summer sea ice was attributed to a warming Southern Ocean, a weakened stratospheric vortex, and atmospheric anomalies. February 2023 marked a record low for Antarctic sea ice (Yu et al. 2021; Ariaan Purich and Doddridge 2023), with 2022 and 2024 tying for the second lowest (NASA 2024). A study of 16 top ice sheet models warned that high greenhouse gases emissions could trigger significant Antarctic ice sheet melt, raising sea levels by up to 6.9 m by 2300, with 40% predicting West Antarctic Ice Sheet collapse by 2300 (Hélène Seroussi et al. 2024).

Both polar oceans, acting as carbon sinks, have seen a 30% rise in ocean acidification since 1850 (Dupont and Pörtner 2013), which is expected to triple by 2100, impacting marine species and ecosystems (IPCC 2023). The oceans absorb around 30% of the excess anthropogenic atmospheric carbon dioxide (CO_2_) emissions (National Oceanic and Atmospheric Administration 2020), leading to ocean acidification. Ocean acidification is particularly high in the Arctic with rates three to four times higher than in other ocean basin from 1994 to 2020 (Qi et al. 2022), partly driven by transport of CO_2_ with ocean currents travelling northwards into the North Pacific and Atlantic Oceans (Bellerby 2017). Ocean acidification will also affect the Antarctic and already-fragile coral ecosystems. Ocean acidification threatens the livelihoods of 400 million people worldwide who depend on marine organisms, leading to socio-economic losses and mental health challenges (Dupont and Pörtner 2013).

Recent studies show rising extreme events causing triple exposure to heat, acidification, and low oxygen. Marine heatwaves combined with deep-water intrusion amplify these effects. In 2019, 20% of the Gulf of Alaska’s shelf bottom water experienced such events, severely impacting species across trophic levels (Hauri et al. 2024).

These polar-driven changes may weaken the jet stream (Barnes and Screen 2015), slow down ocean currents (e.g. the Labrador-Irminger Sea Convection and Atlantic Meridional Overturning Circulation [AMOC]), causing erratic weather patterns and increasing the frequency of global extreme weather events (such as heatwaves, severe cold snaps, floods, droughts, and wildfires), threatening air quality and food and water safety and security worldwide (Overland 2022). By the end of the century, even with rapid decarbonisation, polar regions will be drastically altered, with diminished sea ice and land snow, shrinking glaciers, and habitat shifts, all contributing to global environmental and health impacts through interconnected feedback loops (IPCC 2019).

### Regional direct and indirect health risks in the Arctic

Around four million people live in the Arctic, of whom 10% are Indigenous groups including but not limited to the Inuit, Sámi, Chukchi, and many others across the region (Arctic Council 2024). These communities already face high rates of chronic conditions, mental health issues, food insecurity and socioeconomic challenges, all of which are exacerbated by ongoing environmental changes and insufficient healthcare access.

#### Direct regional risks in the Arctic

Arctic sea ice loss and permafrost thaw heighten human health risks for Arctic communities by destabilizing infrastructure such as buildings, roads, and pipelines. These changes also worsen coastal erosion and flooding, resulting in land loss, water and soil contamination, and a rise in waterborne and other infectious diseases, all of which impact physical and mental well-being (Liew et al. 2022; Gartler et al. 2025).

The release of harmful biological, chemical (mercury) and radioactive substances from thawing permafrost contaminates water, soil, and food chains. These pollutants, historically trapped in ice and soil, originate from both natural sources and long-range industrial emissions that have accumulated in the Arctic through atmospheric and oceanic transport. Arctic communities (Greenland and Nunavik, Faroe Islands, Sámi regions in Northern Norway, Lapland in Northern Finland, Northern Russia, Circumpolar Russia) dependent on fish and marine mammals, face heightened physical health and mental well-being risks, including neurological disorders, cognitive and motor dysfunctions, immune impairments, and lower birth weights (CORDIS 2015; AMAP 2021; Basu et al. 2022; AMAP 2024).

Moreover, permafrost thaw may release dormant viruses e.g. the 1918 influenza virus (Alempic et al. 2023). Some of these viruses can be harbored in long-buried animal carcasses. Permafrost thaw may also release the dormant bacteria, Anthrax spores, through reindeer herds that are a food source for Arctic communities. Ingestion and inhalation of these spores cause fever, muscle aches, chest pain, shortness of breath, and can be fatal without treatment (Hueffer et al. 2020; Ezhova et al. 2021). Furthermore, contaminated food and water chains increase the community’s exposure to antibiotic-resistant bacteria and undiscovered viruses, posing new zoonotic and epizootic disease outbreak risks (Stella et al. 2020; Miner et al. 2021; Andersen-Ranberg et al. 2024).

The frequency of Arctic wildfires tripled, and the burnt area expanded 2.6 times from 2001–2010 to 2011–2020 (Zhu et al. 2023a, b). This has led to chronic diseases risks from PM2.5 air pollution exposure, while also impacting ecosystem dynamics. In Alaska, many rural communities face multiple days each year with air quality deemed unhealthy due to the impact of wildfires (Grigorieva 2024).

#### Indirect regional risks in the Arctic

Ocean warming and acidification, as well as compound extreme events, poses health risks for Arctic communities by imposing new stressing conditions, which is challenging key foundational and provisional species (e.g. oysters, clams, shrimp, caribou, berries, mushrooms and several fish species) (AMAP 2018, 2024. This reduces the sustainability of polar ecosystems (Macias-Fauria and Post 2018) and the quantity and quality of sea and other traditional food for local and export use. Reduced food provision and security negatively affects mental health, and contributes to chronic diseases (e.g. hypertension, malnutrition, miscarriages, kidney failure, cardiovascular issue, lung and bladder cancer, neurovegetative disorders and obesity) (Falkenberg et al. 2020; Bogdanova et al. 2021; Abass et al. 2024).

In the Arctic, melting ice and warmer conditions are causing earlier emergence and northward spread of biting insects such as mosquitoes, blackflies, warble flies, and botflies. These shifts are increasing disease risks (parasitic infections, zoonotic diseases, allergic infections) across northern Canada, Greenland, and Siberia, disrupting ecosystems and Indigenous livelihoods (Koltz and Culler 2021).

Already under-resourced and under-supported by national governments, Indigenous populations are particularly at risk, facing the loss of ancestral lands and cultural traditions, decreased hunting and fishing opportunities, and forced relocations from climate change and ocean acidification impacts. These changes severely affect their mental well-being. Isolation from traditional lands disrupt cultural practices, leading to anxiety, depression, malnutrition, social tension, suicides, and identity loss (Lebel et al. 2022; Mardikian and Galani 2023; Ayeb-Karlsson et al. 2024).

### Direct global health risks

#### Sea level rise and health

Sea level rise due to melting polar ice sheets and glaciers, particularly from Greenland and West Antarctica, intensifies coastal hazards worldwide. Rising sea levels increase the salinity of groundwater, contaminating drinking water sources, impeding agricultural growth, and leading to chronic diseases as well as communicable diseases (e.g. diarrhoea and skin conditions) (Liu and Liu 2014; Talukder et al. 2023).

By 2050, 41 nations across every continent (excluding Antarctica) are projected to experience inland saltwater intrusion extending at least 1 km (Mueller et al. 2024). Models predict a 0.5psu (practical salinity unit) increase in groundwater salinity for every 0.2 m of sea-level rise (Liu and Liu 2014). The standard range for normal groundwater is less than 0.5psu (1 g of salt per liter of water), thus levels could double. This salinity rise has also been linked to a range of health challenges, notably pregnancy complications such as pre-eclampsia and postpartum infant morbidity (Shammi et al. 2019).

Countries such as India, China, Bangladesh, and the Netherlands are particularly vulnerable, with 900 million people living in low-lying coastal areas (United Nations 2023). Rising sea levels increase the salt concentration in aquifers, the underground layers from which groundwater is extracted. This heightened salinity makes the sediments and rocks within the aquifer more porous, causing them to release arsenic into the water. This arsenic release contaminates groundwater and freshwater sources during floods; and poses additional health risks, increasing the likelihood of a range of chronic diseases including cancers (Izaditame et al. 2022; World Health Organization 2022).

Rising sea levels also affect coastal agriculture and fisheries, particularly rice production in countries such as Bangladesh, Japan, Taiwan, and Vietnam. This disrupts traditional diets, contributing to malnutrition and an increased risk of chronic diseases (Semba et al. 2021; Kruger et al. 2022). Flooding from sea-level rise has also been associated with outbreaks of waterborne diseases (e.g. cholera), with impacts seen in diverse regions globally such as West Africa and Southeast Asia (Jung et al. 2023).

#### Extreme weather and polar change

Arctic warming weakens the jet stream, causing prolonged extreme weather specifically in northern and mid-latitude regions, while Antarctic ice melt alters ocean currents, intensifying storms and shifting rainfall patterns (Coumou et al. 2018; Walsh et al. 2020; Francis and Vavrus 2021).

Polar-driven changes in atmospheric patterns and temperatures can intensify hurricanes, typhoons, cyclones, marine heatwaves and resultant flooding, all of which can lead to injuries, fatalities, displacement, and infrastructure loss. Hurricanes also are associated with an increase in chronic diseases including decreased mental health (Waddell et al. 2021). Flooding from cyclones and hurricanes contaminate portable water sources, compromising sanitation and hygiene practices which can lead to an increase in diseases (e.g. cholera, bacterial infections related to *Vibrio vulnificus*, intestinal pathogens, Leptospirosis) (Drake et al. 2023; Huang et al. 2024a, b; Poulakida et al. 2024). Furthermore, there is robust evidence that cholera and other Vibrio species are expanding into higher latitudes (Sweden, Finland, Subarctic regions, Vancouver Island, Canada, Northern Europe), potentially reaching Arctic regions due to warmer sea surface temperatures driven by polar change-induced marine heatwaves. (Trinanes and Martinez-Urtaza 2021; Vezzulli 2022).

Flooding, exacerbated by polar ice melt, leads to immediate deaths, injuries, waterborne diseases, and long-term respiratory problems from mold (Lee et al. 2020). Flooding caused over 100,000 pregnancy losses annually across 33 countries in South and Central America, Asia, and Africa, from 2010 to 2020 (He et al. 2024). In Nepal, flood and landslide deaths have surged, extreme rainfall increases flood mortality by 33% and landslide mortality by 45%, largely due to polar warming driven intensified monsoon over South Asia (Chapagain et al. 2024). Rising temperatures and increased frequency and severity of natural disasters activates outbreaks of fungal infections by creating weather conditions suitable for the fungi (e.g. *Candida auris*) to thrive longer, especially impacting vulnerable populations (Lindsey Konkel Neabore 2024).

Droughts, worsened by changes in polar weather patterns, heighten risks of malnutrition, respiratory issues, waterborne diseases (e.g. *E. coli* and cholera), and airborne diseases (e.g. coccidioidomycosis [Valley fever]) (Stanke et al. 2013). In 2023, an extreme drought lasting at least one month affected 48% of the global land area; and hotter, drier weather fueled more sand and dust storms, exposing 31% more people to dangerously high levels of infected particulate matter (Romanello et al. 2024).

As permafrost thaws, wildfires in northern regions like Siberia and Canada become more frequent, degrading air quality and releasing fine particulate matter (PM2.5) air pollution, contributing to an estimated 25,000–55,000 premature deaths annually in surrounding Arctic and non-Arctic nations (e.g. China) due to transboundary pollution (Johnston et al. 2012; Silver et al. 2023). These wildfires also cause and exacerbate respiratory and cardiovascular issues, pregnancy complications, and increased stress (Alpo Vuorio et al. 2023).

Furthermore, warming induced polar physical changes such as sea and land ice loss can trigger compound extreme weather events through mechanisms such as atmospheric teleconnections and altered moisture transport patterns (Zhu et al. 2023a, b; Su et al. 2024). These compound extreme weather events (e.g., heatwaves and droughts occurring together; a hurricane followed by heavy rainfall and flooding; e.g., wildfires triggered by extreme heat and strong winds) can increase health risks discussed above by 64%, specifically for endocrine diseases, increasing the burden on healthcare resources (Huang et al. 2024a, b).

#### Extreme heat, extreme cold and polar change

Polar physical changes significantly increase the frequency of extreme heat events. Early studies suggest that the seasonally ice-free Arctic may be contributing to a rise in the frequency of severe El Niño episodes, leading to worsening heatwaves especially across tropical areas (Liu et al. 2022a, b). Rising temperatures elevate acute and chronic diseases (Kenny et al. 2014), with a 2.1% rise in cardiovascular mortality per 1 °C increase (approximately 376,000 additional deaths worldwide) (Liu et al. 2022a, b). An increasing concern is heat stress nephropathy, a form of chronic kidney disease (CKD) from repeated heat exposure and dehydration, now emerging globally, linked to rising temperatures (Glaser et al. 2016). Heat stress threatens agriculture, potentially accounting for 60% of global working hours lost by 2030, impacting the physical (CKD) and mental well-being of outdoor workers (Nerbass et al. 2017; Murphy et al. 2023).

In 2023, extreme heat brought an aggregate increase of 50 additional days of health-threatening high temperatures globally with significant regional variations, contributing to a 167% surge (28,390 additional deaths) in heat-related deaths among people over 65 compared to the 1990s. Moreover, high temperatures led to 6% more sleep loss than the 1986–2005 average globally (25–30 min less sleep per night), harming physical and mental health (Romanello et al. 2024). Higher temperatures are linked to increased preterm births, with each 1 °C increase in mean temperature being associated with a 1% rise in early-term births (approximately an additional 134,000 preterm births worldwide) (Darrow et al. 2024), particularly affecting at risk countries with limited data on heat and health records (Thompson et al. 2023). For example, Shanmugam Rekha et al. (2023) found heat stress doubled the risk of miscarriage in pregnant women in India. Furthermore, heat stress can damage the gut lining, allowing digestive enzymes to leak into the body and cause serious health problems including include sepsis, inflammation, multi-organ failure, and even death (Fung et al. 2021).

Similarly, extreme cold events can occur when the polar vortex weakens, allowing cold Arctic air to spill into lower latitudes. This disruption in atmospheric circulation causes unusually harsh cold weather in regions that typically do not experience such extremes. These extreme cold events are linked to increased mortality, particularly from cardiovascular and respiratory causes, and have significant health implications for the elderly and those with pre-existing conditions. (Analitis et al. 2008; Zhang et al. 2021).

### Indirect global health risks

#### Vector-borne diseases and polar change

Rising temperatures in the Arctic due to Arctic amplification are contributing to the emergence and spread of zoonotic and vector-borne diseases, (e.g., vibriosis, tularemia, and tick-borne illnesses) in regions such as Sweden and Russia (Waits et al. 2018). However, the effects of physical polar change are not limited to the Arctic; studies indicate that increased warming is altering the distribution of vector-borne diseases globally. For example, (Chemison et al. 2021), investigated how temperature variations driven by rapid melting of the Greenland ice sheet impact climate and thus malaria transmission in Africa.

Warmer temperatures are enabling mosquitoes and other vectors to thrive in new regions, driving diseases such as malaria to higher altitudes in Colombia and Ethiopia, and facilitating outbreaks of flaviviruses dengue, chikungunya, Japanese encephalitis and West Nile virus in Europe, Asia, Australia, Africa, and Latin America (Daep et al. 2014; García‐Carrasco et al. 2024). By 2050, dengue is projected to expand into new areas such as Amsterdam, Berlin, London, and Stockholm in Europe, as well as parts of North America, Central Africa and East Asia, due to rising temperatures and increasing urbanization (Ebi and Nealon 2016).

Furthermore, research shows that milder winters followed by sudden cold snaps with less snow cover in subarctic Finland and Sweden lead to increased outbreaks of nephropathia epidemica ([NE] a form of hantavirus infection), a rodent-borne disease caused by the Puumala virus (Ma et al. 2021). The disease is associated with health conditions such as fever, kidney issues, and flu-like symptoms. The bank vole (a small rodent found across Europe and parts of Asia), which carries the virus, usually shelters under snow, but when snow cover is poor, they move indoors for warmth, increasing human exposure.

In North America, climate change is linked to earlier and longer Lyme Disease seasons and the northward spread of Lyme-carrying ticks into Canada (Rocklöv and Dubrow 2020). Additionally, temperature changes are causing the rise of rare and lethal mosquito-borne diseases, (e.g. Eastern equine encephalitis [EEE]), and leading to their incursion into new areas in the U.S. (Tang et al. 2021).

#### Food and water borne diseases and polar change

Polar warming and rising ocean temperatures drive changes in the AMOC, disrupting global precipitation and temperature patterns. This impacts agricultural productivity, leading to a reduction in crop yields (e.g. wheat, maize, and rice) (Fodor et al. 2017), increasing the prevalence of malnutrition-related chronic diseases (Shewry and Hey 2015; Jain 2022). This directly affects subsistence farmers, who rely on natural water sources and locally grown food. When combined with extreme heat, food and water borne diseases weaken farmers, reducing their ability to work and further lowering agricultural productivity while increasing disease prevalence.

As discussed earlier, polar change intensifies extreme weather events, including flooding and droughts, which can negatively impact water quantity and quality (Woodward et al. 2016), food security, electricity, communications, and other infrastructure, depending on the degree of preparation and the capacity of health systems. This situation may increase the spread of food and waterborne illnesses (e.g. campylobacteriosis, salmonellosis, cholera, and typhoid), particularly in low- and middle-income countries with poor infrastructure and sanitation services (Semenza et al. 2012; Fenta 2019; Anas et al. 2021). According to the WHO 2023 Global Cholera statistics report, there was a 71% increase in deaths and a 13% rise in reported cholera cases compared to 2022 (World Health Organization 2024).

In 2023, an additional 151 million people faced moderate or severe food insecurity due to a combination of lower agricultural yields from extreme weather events and increased global population compared to the period between 1981 and 2010, risking malnutrition (Romanello et al. 2024). Africa (Sahel region) currently has the highest prevalence of food-borne illnesses (Acosta et al. 2024), followed closely by Southeast Asia (Cissé 2019).

#### Mental health and polar change

As already discussed, polar physical changes are intensifying extreme weather events, food and water scarcity, and ecological disruptions, all of which have significant mental health consequences. Research on the specific mental health impacts of polar physical changes on global regions remains limited. However, the societal costs of the known climate-related mental health disorders are projected to reach $47 billion annually by 2030 and $537 billion by 2050 under the assumption that increasing climate-related events, population growth, and greater exposure to stressors will raise the incidence of mental health disorders (Kumar et al. 2023).

Rising temperatures, especially beyond 3 °C, are linked to increases in anxiety, depression, and suicide rates, with a 2% increase in mental health problems (approximately 9.4 million additional cases) associated with the cumulative effect of a 1 °C temperature rise over a five-year period, globally with regional variability (Obradovich et al. 2018). Heat exacerbates psychiatric conditions, and contributes to hospital admissions for mental health disorders, during extreme heat events, for at risk populations or those with existing mental health disorders.

Extreme weather events and gradual changes in climatic conditions such as rising temperatures and reduced air quality (Clayton 2021), intensified by polar changes, cause psychological trauma and long-term mental health conditions (e.g. Post-Traumatic Stress Disorder [PTSD]). Displacement due to loss of homes, lands, livelihoods and forced migration further strains survivors, contributing to insecurity and despair (Cianconi et al. 2020; Lawrance et al. 2022; White et al. 2023).

## A conceptual framework linking polar change to global and regional health impacts

As highlighted earlier, polar regions are rapidly transforming, potentially triggering feedback loops and tipping cascades. These polar tipping points have significant health implications both locally and globally. Environmental changes (e.g. sea ice loss, permafrost thaw, ocean acidification, and rising sea levels) activate various health risk pathways that impact populations worldwide. Based on our scoping review of the existing literature, Fig. 1a, presents the health risks associated with polar change.

Research has used a variety of approaches to understand the impact of polar change on specific health risks, particularly at the regional level, including changing disease patterns and food and water security risks, alongside regional vulnerability assessments and adaptation strategies. However, a more integrated, transdisciplinary approach remains lacking. Figure 1a and b together illustrate how health risks from polar change span regional and global scales and involve both direct and indirect pathways, from polar physical changes. Yet, according to the scoping review results, the current research often remains siloed by discipline and geographic focus. This highlights the need for transdisciplinary research as a critical cross-cutting theme to connect polar science with public health; and to examine the direct and indirect global health risks from current rates of polar change; and further, to estimate implications of potential non-linear changes if polar tipping points are triggered in terms of unstoppable melt of polar ice sheets, abrupt permafrost thaw and methane release, and changes to ocean dynamics (both in terms of ocean acidification and the weakening of the Labrador Sea subpolar gyre).

Existing interdisciplinary frameworks that could usefully explore global health risks from potential polar tipping points and ongoing polar change (Fig. 1a) include: The Lancet Countdown, the Arctic Monitoring and Assessment Programme of the Arctic Council, One Health, and the Global Burden of Disease (GBD) study. These frameworks all attempt to integrate health and environmental data. Initiatives such as Planetary Health Alliance, and Circumpolar Health Research Network (CircHNet) emphasize incorporating Indigenous knowledge for holistic assessments (Mackenzie and Jeggo 2019; AMAP 2021; Murray 2022; Romanello et al. 2022).

Polar tipping points in climate systems are often underestimated in scientific projections because climate models rarely account for multiple interacting stressors, which are complex. As policies frequently aim to operate near these thresholds, such underestimations can lead to severe socio economic and health risks. At the same time, the inclusion of polar change into future climate-health impact assessments faces challenges such as fragmented research, poor interdisciplinary collaboration, inconsistent environmental and health data, and uncertainties inherent in polar change and modeling these dynamics within global climate models. For example, Coupled Model Intercomparison Project Phase 6 (CMIP6) (which are Earth system models) tend to underestimate the observed rates of ice melt in Greenland and the West Antarctic, and do not fully represent some processes such as the effects of abrupt permafrost thaw (including wildfire impacts and the burning of organic soils on permafrost) and the progression of ocean acidification.

Furthermore, calculating the health risks from climate tipping points is challenging due to the emerging nature of tipping point science and the lack of specialised geo-physical models and emulators to fully quantify broad range of impacts from abrupt shifts of certain climate tipping points (Terpstra et al. 2024) (see Table S5).

The lack of integration between health, food and water security, and climate change research hinders the development of comprehensive indicators. Barriers such as data fragmentation, release of health and weather records separately, incompatible data formats and a lack of agreed metrics; these all slow down the timely analysis and policy responses, especially for under-resourced communities (Naumova 2024) (See Fig. 1b). Moreover, the emerging physical and mental health risks to at risk communities globally, and the profound regional effects on the health and cultural lifestyles of Arctic Indigenous, strain already vulnerable medical and public health systems (Bogdanova et al. 2021).

## Agenda setting: Implications for future research

Figure 1a offers a conceptual framework that can help guide the development of a system based iterative health risk management framework that accounts for the risks and impacts of physical polar changes and polar tipping points (Rocklöv et al. 2021). A renewed interdisciplinary research agenda is needed to develop robust health frameworks addressing both direct and indirect health risks from polar changes including analysis of spatial and temporal distribution of risks, that assess all components of risks (hazard, exposure and vulnerability) (Morris et al. 2017).

Moreover, inclusion of the effects of under-represented polar tipping points in mainstream climate impact assessments and the analysis of corresponding health risks are necessary to accurately project future health impacts. Integrating climate data and more specifically polar science data (both observational and model outputs) into health assessments can refine our understanding of systemic dynamics across fields.

Current studies inadequately link polar changes to human health and wellbeing outcomes on a global and regional level. There is limited exploration of the interplay between ocean health, mental health and physical human health including climate-related diseases (Fleming et al. 2024). Although the number of mental health studies is limited, existing evidence already shows that climate-related stress, anxiety, and trauma are emerging concerns, highlighting the urgent need for more systematic research on mental health impacts. Existing health indicators capture the complexities of multi-hazard interactions and socio-economic disparities in only a very limited capacity (Di Napoli et al. 2022). The absence of harmonized health indicators remains a major barrier to international collaboration and limits the comparability of health outcomes across regions. Research gaps highlight insufficient data integration and the under-representation of data from under-resourced regions and at local scale, especially in low- and middle-income countries (LMICs).

Future research can further delineate the dynamics between polar change and global and regional health risks. Such an agenda requires greater data accessibility, and the fostering of local, regional and global partnerships to capture geographically differentiated health risks and provide strong region-specific health risk attributions. In addition, AI methods could also be applied to underutilized global datasets, helping to extract valuable health and environmental information and reduce duplication by making better use of existing data resources. Importantly, research must also account for the critical role of development choices, such as poverty alleviation, investments in universal healthcare, education, and resilient infrastructure, which fundamentally shape community vulnerability and adaptive capacity. Enhancing healthcare resilience and achieving equitable health outcomes in the face of polar-induced health challenges depends not only on environmental monitoring but also on addressing these underlying social and economic determinants. Moreover, future research should focus on generating granular local health data in Arctic and other under resourced communities and explicitly linking infrastructure vulnerabilities to polar physical changes to better inform targeted and socially just adaptation strategies.

Future research can usefully explore how polar change dynamics can be integrated into existing health frameworks such as the Driver, Pressure, State, Exposure, Effect, and Action (DPSEEA), the Climate-Adaptive System Based Framework, World Health Organization’s Framework for Health Quantification and Operational Framework for building climate resilient health systems, the Integrated Climate Change and Health Indicator System Framework, the Driver-Pressure-State-Impact-Response (DPSIR) Framework and Arctic specific health frameworks (Arctic One Health framework, Iceberg framework and Co-production of knowledge in the context of Arctic research) (see Table S6). Finally, shifting toward predictive, health-centered metrics (tools that use data to project future health outcomes) and ensuring interoperability and alignment between public health data and environmental datasets can enhance collaboration between polar and climate science and health sectors.

## Conclusion

Physical polar change and potential polar tipping points (e.g. permafrost thaw, ice sheet collapse, and sea ice loss) drive abrupt climate changes with severe global health impacts, extreme weather events, heat stress, air quality, water quality and quantity, food supply and safety, and vector distribution and ecology, disease dynamics and sea level rise. Under resourced communities worldwide, with a higher burden on low- and middle-income countries, face heightened risks due to weak healthcare systems and other essential infrastructure.

Addressing these challenges requires interdisciplinary research to assess health risks, focusing on food and water systems, socio-economic factors, and mental health, priority areas identified in the scoping review as being particularly sensitive to climate and polar physical changes and based on their disproportionate impact on under-resourced communities. Polar health research must overcome territorial approaches, engaging with ecological systems beyond governance boundaries to address interconnected health and environmental challenges. Global funding, ethically governed health–environment data integration, comprehensive polar-health risk assessment, and stronger policy frameworks are needed to enhance healthcare resilience and adaptive capacity. Immediate international collaboration grounded in transdisciplinary partnerships between health and environmental sciences is crucial to mitigate the health impacts of accelerating climate change.

## Supplementary Information

Below is the link to the electronic supplementary material.

Supplementary file1 (XLSX 86 KB)

Supplementary file2 (PDF 409 KB)

## Footnotes

1 (https://www.rayyan.ai/).
