# Supplementary material for: A framework for assessing global health impacts of polar change: An urgent call for interdisciplinary research
Source: Ambio. 2025 Nov 7;55(3):529–45. doi: 10.1007/s13280-025-02255-0 (PMC12868344; doi:10.1007/s13280-025-02255-0)

Supplementary Information for

A framework for assessing global health impacts of polar change: An urgent call for interdisciplinary research

Authors:

Netra Naik , Karol Bot ,Gail Whiteman*, Lora E. Fleming, Karyn Morrissey, Richard Bellerby, Sam Dupont, Dmitry Yumashev, Susana Hancock, Brendan M. Rogers, Kristie L Ebi, Joacim Rocklöv.

Correspondence: Gail Whiteman

Email: g.whiteman@exeter.ac.uk

This Pdf includes Tables S1 to S6 and Fig S1

Table S1. Research question and keywords

**Research question**
"Can human health risks be linked to physical polar changes (PPC), and if so, what are the manifestations of these impacts now and in the future?"<br><br>Key words:
**Polar changes**
Polar change, Polar ice melt, permafrost thaw, ocean acidification, Atlantic Meridional Overturning Circulation (AMOC), ocean currents, polar tipping points, labrador irminger sea/spg convection
AND
**Health risks**
Anthrax, chronic diseases, neurological and cognitive disorders, respiratory diseases birth related defects, vector borne diseases, foodborne diseases, waterborne diseases, Cholera, Dengue, Malaria, West Nile Virus, anxiety, depression, anger, stress, distress, trauma, PTSD, suicide, well-being, Mental Illness, Mental Health, mental wellbeing, Mental Disorder, psychological impacts.
AND
**Global climate change consequences**
climate change, greenhouse gas, fossil fuels, global warming, global climate change, extreme weather events, heat, flooding, drought, storm, sea level rise, forest fires, wildfires, storms, hurricanes, air quality, water quality, soil quality, food quality, precipitation.

Table S 2. Details of database searches performed.

Database	Search strings and Booleans	Filters	Initial Results
1) Pubmed	Polar change AND/OR Health risks AND/OR Global climate change consequences	Publish year: 2009 to 2025<br><br>Text availability: Abstract, Free full text, full text.<br><br>Including associated data<br>Article type: Meta-Analysis, Multicenter Study, Observational study, Periodical Index Review, Systematic review<br><br>Geographic area: global	116 studies were found in this search thread<br>https://pubmed.ncbi.nlm.nih.gov/?term=Polar+change+AND%2FOR+Health+risks+AND%2FOR+Global+climate+change+consequences&filter=simsearch1.fha&filter=simsearch2.ffrft&filter=simsearch3.fft&filter=articleattr.data&filter=pubt.meta-analysis&filter=pubt.multicenterstudy&filter=pubt.observationalstudy&filter=pubt.review&filter=pubt.systematicreview
2) Web of Science	**Search query:** Polar change AND/OR Health risks AND/OR Global climate change consequences<br><br>**Added Keywords:** Polar change, Polar ice melt, permafrost thaw, ocean acidification, Atlantic Meridional Overturning Circulation (AMOC), ocean currents, polar tipping points, labrador irminger sea/spg convection ***AND*** Anthrax, chronic diseases, neurological and cognitive disorders, respiratory diseases birth related defects, vector borne diseases, foodborne diseases, waterborne diseases, Cholera, Dengue, Malaria, West Nile Virus, anxiety, depression, anger, stress, distress, trauma, PTSD, suicide, well-being, Mental Illness, Mental Health,	Publish year: 2009 to 2025<br><br>Text availability: Abstract, Free full text, full text.<br><br>Article type: Meta-Analysis, Multicenter Study, Observational study, Periodical Index Review, Systematic review.<br><br>Geographic area: global	6 studies were found in this search thread<br>https://www.webofscience.com/wos/woscc/summary/21b79efe-2914-4fc0-821a-f4f9abe0367f-016b0859f1/relevance/1

	mental wellbeing, Mental Disorder, psychological impacts ***AND*** climate change, greenhouse gas, fossil fuels, global warming, global climate change, extreme weather events, heat, flooding, drought, storm, sea level rise, forest fires, wildfires, storms, hurricanes, air quality, water quality, soil quality, food quality, precipitation.		
3)Scopus	Polar change AND/OR Health risks AND/OR Global climate change consequences	Publishing year: 2009 to 2025<br><br>Subject area: Earth and Planetary Sciences, Medicine, Social Sciences, Multidisciplinary<br><br>Document type: Article, Review<br><br>Language: English<br><br>Additional keywords: Climate change, Extreme event, Air pollution, allergic Asthma, allergic disease, allergic rhinitis, health risks, water supply, warming, vulnerable population, subsidence, soils, respiratory tract disease, public risks, precipitation extremes, polar vortex, pathogenesis, particulate matter ,jet stream, increased temperature, humidity, human, health, extreme weather events, climate change and respiratory disease, chronic obstructive lung disease, cascading risks, cardiovascular disease, troposphere,	4 studies were found in this search thread. https://www-scopus-com.uoelibrary.idm.oclc.org/results/results.uri?st1=Polar+change%2C+Health+risks%2C+Global+climate+change+consequences&st2=Polar+change%2C+Polar+ice+melt%2C+permafrost+thaw%2C+ocean+acidification%2C+Atlantic+Meridional+Overturning+Circulation+%28AMOC%29%2C+ocean+currents%2C+polar+tipping+points%2C+labrador+irminger+sea%2Fspg+convection&yearFrom=2009&yearTo=Present&s=%28TITLE-ABS-KEY%28Polar+change%29+AND+TITLE-ABS-KEY%28Human+health+risks%29+AND+TITLE-ABS-KEY%28Extreme+weather+events%29%29&limit=200&origin=resultslist&sort=plf-f&src=s&sot=b&sdt=cl&sessionSearchId=555b9292b7c308b87823abd9034b410b&cluster=scoexactkeywords%2C%22Climate+Change%22%2Ct%2C%22Health+Risks%22%2Ct%2C%22Extreme+Event%22%2Ct%2C%22Air+Pollution%22%2Ct%2C%22Water+Supply%22%2Ct%2C%22Water+Conservation%22%2Ct%2C%22Warming%22%2Ct%2C%22Vulnerable+Population%22%2Ct%2C%22Subsidence%22%2Ct%2C%22Soils%22%2Ct%2C%22Soil+And+Water+Conservation%22%2Ct%2C%22Respiratory+Tract+Disease%22%2Ct%2C%22

		atmosphere, stratosphere, risk assessment, review, photochemical smog, high risk population, global warming, disaster, Cmip6,<br><br>Geographic area: global	Respiratory+System%22%2Ct%2C%22Public+Risks%22%2Ct%2C%22Precipitation+Intensity%22%2Ct%2C%22Precipitation+Extremes%22%2Ct%2C%22Polar+Vortex%22%2Ct%2C%22Pathogenesis%22%2Ct%2C%22Particulate+Matter%22%2Ct%2C%22Jet+Stream%22%2Ct%2C%22Increased+Temperature%22%2Ct%2C%22Humidity%22%2Ct%2C%22Human%22%2Ct%2C%22Health%22%2Ct%2C%22Extreme+Weather+Events%22%2Ct%2C%22Climate+Change+And+Respiratory+Disease%22%2Ct%2C%22Chronic+Obstructive+Lung+Disease%22%2Ct%2C%22Cascading+Risks%22%2Ct%2C%22Cardiovascular+Disease%22%2Ct%2C%22Allergic+Rhinitis%22%2Ct%2C%22Allergic+Asthma%22%2Ct%2C%22Allergic+Disease%22%2Ct%2C%22Wind+Velocity%22%2Ct%2C%22Uncertainty+Analysis%22%2Ct%2C%22Uncertainty%22%2Ct%2C%22Troposphere%22%2Ct%2C%22Stratosphere%22%2Ct%2C%22Risk+Reduction%22%2Ct%2C%22Risk+Assessment%22%2Ct%2C%22Review%22%2Ct%2C%22Photochemical+Smog%22%2Ct%2C%22Ozone%22%2Ct%2C%22High+Risk+Population%22%2Ct%2C%22Global+Warming%22%2Ct%2C%22Global+Scale%22%2Ct%2C%22Freedom+Of+Movement%22%2Ct%2C%22Disaster%22%2Ct%2C%22Coupled+Model+Intercomparison+Project+Phase+6%22%2Ct%2C%22Cmip6%22%2Ct%2C%22Climate+Models%22%2Ct%2C%22Atmosphere%22%2Ct%2C%22Adequate+Standard+Of+Living%22%2Ct%2Bscosubjabbr%2C%22EART%22%2Ct%2C%22MEDI%22%2Ct%2C%22MULT%22%2Ct%2C%22SOCI%22%2Ct%2Bscosubtype%2C%22ar%22%2Ct%2C%22re%22%2Ct%2Bscolang%2C%22English%22%2Ct
4)Science direct	**Search query:** Polar change AND/OR Health	Publishing year: 2009 to 2025	28 studies were found in this search thread:

	risks AND/OR Global climate change consequences<br><br>**Added Keywords:** Polar change, Polar ice melt, permafrost thaw, ocean acidification, Atlantic Meridional Overturning Circulation (AMOC), ocean currents, polar tipping points, labrador irminger sea/spg convection ***AND*** Anthrax, chronic diseases, neurological and cognitive disorders, respiratory diseases birth related defects, vector borne diseases, foodborne diseases, waterborne diseases, Cholera, Dengue, Malaria, West Nile Virus, anxiety, depression, anger, stress, distress, trauma, PTSD, suicide, well-being, Mental Illness, Mental Health, mental wellbeing, Mental Disorder, psychological impacts ***AND*** climate change, greenhouse gas, fossil fuels, global warming, global climate change, extreme weather events, heat, flooding, drought, storm, sea level rise, forest fires, wildfires, storms, hurricanes, air quality, water quality, soil quality, food quality, precipitation.	Article type: Review articles, Research articles, data articles, discussion, mini reviews<br><br>Subject areas: Environmental sciences, Earth and Planetary Sciences, Social Sciences, Agricultural and Biological Sciences<br><br>Publication title: Science of the Total Environment, Climate Risk Management, Earth Science Reviews, Environmental International, Journal of Hydrology, Atmospheric Environment, Ocean and Coastal Management, Marine Pollution Bulletin, Atmospheric Research, Journal of Environmental Management, Sustainable Cities and Society, Marine Policy, Urban Climate, The Lancet	https://www.sciencedirect.com/search?qs=Polar%20physical%20change%20AND%20Human%20Health%20risks%20AND%20Global%20climate%20change%20consequences%20AND%20extreme%20weather%20events%20AND%20sea%20level%20rise&date=2009-2025&articleTypes=REV%2CFLA%2CDAT%2CSSU%2CDIS&publicationTitles=271800%2C271741%2C272592%2C280276%2C283182%2C271824%2C271825%2C271842%2C271881%2C271798%2C271816%2C282307%2C271074%2C271763&subjectAreas=2300%2C1900%2C3300%2C1100&accessTypes=openaccess&show=100
5)Consensus	Polar change, Polar ice melt, permafrost thaw, ocean acidification, Atlantic Meridional Overturning Circulation (AMOC), ocean currents, polar tipping points, labrador	Publish year: 2009 to 2025<br>Journal rank (Q1-Q4)<br><br>Methodology: Meta-Analysis, Systematic reviews, observational	29 studies were found in this search thread.<br>https://consensus.app/search/polar-change-health-impacts/Vn0VgyC8QCa2i9b5aWhFBw/

	irminger sea/spg convection ***AND*** Anthrax, chronic diseases, neurological and cognitive disorders, respiratory diseases birth related defects, vector borne diseases, foodborne diseases, waterborne diseases, Cholera, Dengue, Malaria, West Nile Virus, anxiety, depression, anger, stress, distress, trauma, PTSD, suicide, well-being, Mental Illness, Mental Health, mental wellbeing, Mental Disorder, psychological impacts ***AND*** climate change, greenhouse gas, fossil fuels, global warming, global climate change, extreme weather events, heat, flooding, drought, storm, sea level rise, forest fires, wildfires, storms, hurricanes, air quality, water quality, soil quality, food quality, precipitation.	studies, literature review, case reports<br><br>Human studies only<br><br>Fields of studies: Environmental studies, geography, geology, medicine, physics, sociology, psychology<br><br>Countries: All	
6)Nature Database	Polar physical change AND Human Health risks AND Global climate change consequences AND extreme weather events AND sea level rise	Publish year: 2009 to 2025<br><br>Article type: all<br>Journal: all<br>Subject: all	41 studies were found in this thread
7)Google scholar search	**Search query:** Polar change AND/OR Health risks AND/OR Global climate change consequences<br><br><br>**Added Keywords:** Polar change, Polar ice melt, permafrost thaw, ocean acidification, Atlantic Meridional Overturning Circulation (AMOC), ocean	Publish year: 2009 to 2025<br><br>Article type: all<br>Journal: all<br>Subject: all	35 studies were found in this thread

	currents, polar tipping points, labrador irminger sea/spg convection ***AND*** Anthrax, chronic diseases, neurological and cognitive disorders, respiratory diseases birth related defects, vector borne diseases, foodborne diseases, waterborne diseases, Cholera, Dengue, Malaria, West Nile Virus, anxiety, depression, anger, stress, distress, trauma, PTSD, suicide, well-being, Mental Illness, Mental Health, mental wellbeing, Mental Disorder, psychological impacts ***AND*** climate change, greenhouse gas, fossil fuels, global warming, global climate change, extreme weather events, heat, flooding, drought, storm, sea level rise, forest fires, wildfires, storms, hurricanes, air quality, water quality, soil quality, food quality, precipitation.		
8) Expert recommended resources	Reports (WHO, AMAP, Lancet), Recommended papers from health and climate change experts	Not applicable	25
			Total initial studies found: 284

Table S3. Inclusion and Exclusion Criteria using PICO categorization:

PICO	Inclusion Criteria	Exclusion Criteria
Population	• All age groups<br>• Neonatal period<br>• All genders/sexual orientations<br>• Arctic Indigenous communities	• N.A
Intervention	• Must relate to climate change, public health and global environmental consequences<br>• Qualitative, quantitative studies, and case reports will be included.<br>• Publication year must be from 2009 onwards.<br>• English language	• Non-English literature<br>• Literature published before the year 2009<br>• Health concerns not linked to environmental change
Comparison	• N.A	• N.A
Outcomes	• Public health issues that can be traced back to change of weather patterns resulting from polar physical changes and polar tipping points<br>• Direct and indirect health risks on Arctic communities rising from infrastructure damage, flooding, permafrost thaw and ocean acidification<br>• Indirect global health risks from increase in frequency of extreme weather events, other categories such as vector-borne diseases, food and water borne diseases, and mental health issues<br>• Direct global health risks from sea level rise such as damage to infrastructure, flooding, increase in salinity	• Public health issues not connected with climate change parameters will be excluded from this study.<br>• Public health issues that cannot be traced back to polar physical changes and polar tipping points will be excluded<br>• Only specific age group or gender focused studies eliminating other vulnerable groups<br>• Studies with overly narrow demographic focus excluding broader vulnerable groups

PICO Categorization: (Singh & Sharan, 2024)

Table S 4. Health Risks from Polar Physical Changes

Polar Physical Changes	Regional Direct and Indirect Health Risks	Direct Global Health Risks	Indirect Global Health Risks
•Loss of sea ice<br>•Loss of land ice<br>•Abrupt permafrost thaw<br>•Changes to Labrador-Irminger Sea convection and AMOC<br>•Sea level rise<br>•Ocean Acidification	Direct Risks<br>• Waterborne diseases and other infectious, impacting physical and mental well-being (Liew et al., 2022; Gartler et al. 2025)<br>• Pathogen and chemical contaminated food, water and soil webs causing Neurological disorders, cognitive and motor dysfunctions, immune impairments, and lower birth weights (AMAP, 2021; Basu, 2022; Cordis, 2013)<br>• Antibiotic resistant bacteria, influenza virus, and undiscovered viruses posing new and rare disease outbreak risks,zoonotic and epizootic disease,Anthrax outbreaks from reindeer herds (Alempic et al. 2023; Stella et al., 2020; Miner et al., 2021; Hueffer et al., 2020; Ezhova et al., 2021;	• Sea level rise, flooding and contaminated drinking water sources: Chronic diseases (e.g. miscarriages, hypertension, kidney failure, and other cardiovascular issues); as well as communicable diseases (e.g. diarrhea, cholera, and skin conditions), (Talukder et al., 2023; Jung et al., 2023; W. Liu & Liu, 2014; Mueller et al. 2024; United Nations, 2023)<br>• Sea level rise and salinity: pregnancy complications such as pre-eclampsia and postpartum infant morbidity (Shammi et al., 2019).<br>• Sea level rise and arsenic levels: lung and bladder cancer, diabetes, hypertension, and neurodegenerative disorders (Izaditame et al., 2021; WHO & IARC, 2022).<br>• Sea level rise and food security: malnutrition and an increased risk of hypertension, kidney failure, and cardiovascular events (Kruger et al., 2022; Semba, 2022)	• Rising temperatures and Vector borne diseases: a) (e.g., vibriosis, tularemia, and tick-borne illnesses) in regions like Sweden and Russia (Waits et al., 2018). b) Malaria transmissionChemison.A *et al.* (2021) c) Lyme disease (Rocklöv & Dubrow, 2020) d) Eastern equine encephalitis (EEE) (Tang et al., 2021) e) nephropathia epidemica (NE) - a form of hantavirus (Ma et al. 2021)<br>f) West Nile virus (José-María García-Carrasco et al. 2024)<br>g) dengue, Chikungunya and West Nile virus (José-María García-Carrasco et al. 2024; Ebi and Nealon 2016)<br>h) Japanese encephalitis, flaviviruses (Daep et al. 2014)<br>• Food and Waterborne diseases: a) Agricultural productivity loss can cause malnutrition-related chronic conditions such as hypertension, diabetes, obesity, and cardiovascular diseases (Shewry & Hey, 2015; Jain, 2022; Cissé 2019; Fodor et al. 2017; Acosta et al. 2024). b) Drought and flooding impact water quality and food security causing (including campylobacteriosis, salmonellosis, cholera, and typhoid); particularly in low- and middle-income countries (Semenza et al., 2012;

	Andersen-Ranberg et al. 2024).<br>• Arctic wildfires: cardiovascular and respiratory diseases (Zhu et al., 2023; Grigorieva, 2024)<br><br>Indirect Risks<br>• Aerosolization of natural toxins, degrading air quality and exacerbating respiratory issues (Falkenberg et al., 2020)<br>• Impact on traditional food habits further exacerbating chronic conditions such as respiratory diseases, hypertension, and obesity (AMAP,2018; Bogdanova et al., 2021, Macias-Fauria and Post 2018; Abass et al. 2024).<br>• Biting insects spreading northward: parasitic infections, zoonotic diseases, allergic infections.(Koltz and Culler 2021)<br>• Isolation from traditional lands disrupts cultural practices, leading to anxiety, depression, social tension,	• Extreme weather (Walsh et al. 2020): a) Prolonged heatwaves cause cardiorespiratory and other diseases, mental health issues, adverse pregnancy and birth outcomes, miscarriage (Jiping Liu et al. 2022;Jingwen Liu et al. 2022; Darrow et al., 2024; Rekha et al. 2023; Thompson et al. 2023; KENNEY et al. 2014) b) Flooding causes waterborne diseases, and long-term respiratory problems from mold, along with mental health impacts such as Post-Traumatic Stress Disorder (PTSD), depression and mortality. (J. Lee et al., 2020; Chapagain et al. 2024; Stanke et al. 2013); Fungal infections (e.g. Candida auris, Valley River), especially (Neabore, 2024); Miscarriage (He et al., 2024) c)Storms, typhoons, hurricanes and cyclones lead to injuries, fatalities, displacement, infrastructure loss, and mental health disorders, particularly PTSD (Francis and Vavrus 2021; Coumou et al. 2018; Waddell et al., 2021); an increase in Cholera,	Fenta, 2019; Anas et al., 2021; World Health Organization, 2024; Woodward et al. 2016) Climate related mental health disorders: anxiety, depression, and suicide rates, psychological trauma and long-term mental health conditions, Post-Traumatic Stress Disorder (Kumar et al., 2023 ; Cianconi et al., 2020 ; Obradovich et al., 2018; Clayton 2021; Lawrance et al. 2022; White et al. 2023).

	suicidal thoughts, and cultural loss (Mardikian & Galani, 2023; Ayeb-Karlsson et al. 2024; Lebel et al. 2022).	vibrio vulnificus infections, intestinal diseases, Leptospirosis (Drake et al., 2023; Huang et al., 2024; Poulakida et al. 2024; Trinanes and Martinez-Urtaza 2021; Vezzulli 2022) d) Wildfires also cause and exacerbate respiratory and cardiovascular issues, pregnancy complications, and increased stress (Vuorio et al., 2023;Johnston et al. 2012; Silver et al., 2023) e) Droughts cause malnutrition, respiratory issues, waterborne diseases (e.g. E. coli and cholera), and airborne diseases (e.g. coccidioidomycosis [Valley fever]) (Romanello et al. 2024)<br>• Extreme heat: a) impact on physical and mental well-being of outdoor workers (Murphy et al. 2023;Nerbass et al. 2017) b) food insecurity and malnutrition (Romanello et al. 2024) c) chronic kidney disease (CKD) (Glaser et al. 2016)<br>• d) gut lining damage, sepsis, inflammation multiorgan failure and death (Fung et al. 2021)<br>• Extreme cold: Cardiovascular and	

		respiratory causes, mortality (Analitis et al., 2008; Zhang et al., 2021)<br>• Compound extreme weather events: endocrine diseases (Su et al. 2024; Huang et al. 2024 ; Xiaoyu Zhu et al. 2023)	

Table S 5. Polar Tipping Points representation in CMIP6 Models

Category of climate tipping elements/abrupt shifts/nonlinear feedbacks	Are the underpinning physical processes included in CMIP6 models?	Are the associated abrupt shifts detectable in CMIP6 models?	Can the resulting impacts on health / agriculture be quantified using current frameworks and statistical approaches?
Category 1: Greenland and West Antarctic ice sheets' decline	No	N.A	No – requires dynamic emulator and DIVA model
Category 2: Abrupt permafrost thaw	No	N.A	Yes – requires dynamic emulator and CMIP6 adjustments
Category 3: More or less certain tipping elements = North Atlantic subpolar gyre; Tibetian Plateau<br><br>Uncertain tipping elements = Indian, West African and South American Monsoons; Antarctic sea ice<br><br>Not tipping elements = Arctic summer sea ice; Arctic winter sea ice; Barents sea ice	Yes	Yes – in some but not all CMIP6 models	Maybe – for example, by comparing between ensembles of CMIP6 models with and without the relevant detectable abrupt shifts. The latter is equivalent to subjectively adjusting probability distributions in multi-model assessments of climate uncertainties. It's not clear what this will add to existing assessments where all CMIP6 models are treated equally
Category 4: Tipping elements = Amazon rainforest; Boreal forests	Yes	No	Unclear – the fact that the underpinning processes are included in CMIP6 models but abrupt shifts are not detectable makes it more difficult to justify introducing emulators

Table S 6. Health Frameworks, Applications, Strengths and Limitations

Health framework: name and description	Applications	Strengths	Limitations
**DPSEEA** (Corvalan et al., 1999).	To assess and monitor human health vulnerability, to aid in the design and targeting of interventions, and measure the effectiveness of climate change adaptation and mitigation activities.	It is the best suited framework for developing Environmental Health Impact Assessments (EHIs), works well for risks associated with environmental pollution.	DPSEEA is less suited for natural hazards or indirect health effects like famine.
Project NODE – Adapted DPSEEA (Boylan et al., 2018).	The framework explores the effects of various exposures on health and well-being, emphasizing the interaction with vulnerability. It aims to support the development of climate change adaptation policies that offer health co-benefits.	The framework clarifies climate change's effects on health, wellbeing, and social vulnerabilities, pinpointing optimal areas for adaptation and response policies.	The framework provides only a qualitative assessment of health impacts and does not quantify vulnerability or health risks.
Climate-Adaptive System-Based (CAS) framework (Talukder et al., 2024).	The framework aids decisions on ecological services or public health interventions to reduce climate change's health impacts. Using empirical data and coupling with simulation models can also help develop multi-sector adaptation policies.	The framework reveals the causal links shaping health outcomes and can also offer a collective narrative of the complex relationship of variables and agents.	The framework's variables are not exhaustive but offer an example based on a hypothetical location, drawing on the authors' experience in Bangladesh and Malawi, where floods and droughts are becoming more frequent due to climate change. Local expertise is needed.
WHO framework for the quantification and economic valuation of health outcomes (WHO, Climate Change and Health (CCH), 2023).	The framework can be used to quantify and forecast health impacts and their economic valuation under scenarios of action and inaction, supporting informed decision-making.	It leverages weather and climate data alongside biophysical and economic statistical models to forecast health impacts over time	Assigning economic values to health outcomes is challenging and can lead to varying results, affecting the perceived cost-effectiveness of climate actions.
Operational framework for building climate	Used by health systems and governments to integrate climate resilience and low-carbon	Comprehensive, flexible, and aligned with global goals, promoting cross-sectoral action	Limited real-world evaluations, insufficient focus on social determinants

resilient health systems (World Health Organization (WHO); Climate Change and Health (CCH), Environment, Climate Change and Health (ECH); 2015)	strategies into public health planning and infrastructure.	and sustainable health system transformation.	like poverty and universal healthcare, and data/resource constraints in low-income settings.
eDPSEEA (Morris et al., 2017 ; Reis et al., 2013)	The framework helps capture the complex links between ecosystem services and human well-being, such as how losing cultural services like recreational fishing affects well-being.	The eDPSEEA framework is effective in capturing complex relationships and identifying broader impacts of ecosystem changes.	Applying the eDPSEEA framework requires strong stakeholder engagement and further refinement for effective policy use.
DPSIR framework (Troian et al., 2021).	The framework outlines the path from driving forces to health impacts and responses, providing a structured approach to environmental issues.	A strength of the framework is its ability to explain the interactions between agriculture and aquatic ecosystems at the watershed level, making it useful for guiding public policy and water resource management decisions.	A limitation of the framework is that it may oversimplify complex environmental issues by using a linear model, which can lead to miscommunication and overlook important ecological feedback loops.
Tangled net concept (Fleming et al., 2019).	The framework focuses on the intricate links between human health and ocean-related activities.	The strength of the tangled net framework lies in its ability to capture complex, interconnected relationships between multiple factors, providing a more dynamic and realistic representation of systems compared to linear frameworks.	The framework needs strong local expertise to effectively address specific contextual factors.
INHERIT Framework (Van Der Vliet et al., 2018).	The INHERIT framework is used across various fields like active transport, energy-efficient housing, green spaces, and food consumption.	The framework supports intersectoral action by identifying unintended health impacts and promoting opportunities to enhance environmental sustainability, health, and equity.	The framework simplifies reality by showing only one feedback loop (impacts to drivers), though multiple loops may exist, enhancing its utility but limiting precision.

The Compass framework (Larsen et al., 2021).	The Compass framework is a conceptual framework to guide the identification of risks and the consequent development of adaptation and mitigation strategies in coproduction with local stakeholders.	The framework balances and integrates scientific assessments and evidence about risk factors with risk perceptions of the affected communities and creates spaces for the coproduction of knowledge and actions	The framework lacks focus on integrating expert and local knowledge in the data-gathering and decision-making process.
Integrated Climate Change and Health Indicator System Framework (Liu et al., 2021)	The framework links climate factors, health impacts, vulnerabilities, and adaptation strategies to assess and improve resilience to climate change.	Transitioning from individual metrics to a system of interconnected indicators enhances the ability to monitor trends, project future scenarios, and assess the effectiveness of interventions.	Coordinating among various institutions with differing priorities can hinder data integration for indicator development and the smooth functioning of the indicator system.
European Centre for Disease Prevention and Control E3 Network (ECDC, 2013)	The network supports early warning systems by identifying climate-driven disease outbreaks, such as vector-borne diseases influenced by temperature changes.	The E3 Network enhances disease forecasting by integrating environmental and epidemiological data into a centralized geospatial system.	Variability in data collection across European countries can lead to inconsistencies in disease risk assessments.
US Centers for Disease Control and Prevention (USCDC, 1992)	Supports early detection of environmental health risks, such as air pollution-related respiratory diseases.	The CDC's National Environmental Public Health Tracking Network integrates environmental and health data, enabling evidence-based public health decisions.	Data gaps and delays can limit real-time surveillance and response effectiveness.
Vulnerability and Adaptation assessments. (IPCC, 1990)	It can be used in climate policy, disaster risk reduction, and infrastructure planning to identify adaptation priorities.	V&A assessments provide a structured, science-based approach to assess climate risks and guide adaptation strategies.	It often relies on uncertain climate projections and lacks standardized methods across regions.
**Arctic specific health frameworks**			
Iceberg Framework (Cueva, et.al; 2021)	The iceberg framework guides Arctic health research by encouraging community-led, participatory approaches that explore both visible health indicators and deeper contextual	It promotes a holistic understanding of health by integrating these often-overlooked social, cultural, and ecological dimensions alongside traditional epidemiological data.	It requires meaningful, long-term community engagement and may be difficult to align with standardized health metrics and

	factors, such as cultural practices, historical experiences, language, social cohesion, and environmental conditions that influence well-being.		top-down policy frameworks.
Arctic One Health Framework (Berner, et.al; 2024)	Integrates human, animal, and environmental health through Arctic Council groups, national projects, and academic networks.	Promotes collaboration, early disease detection, and local capacity building.	Faces fragmented coordination, data gaps on pathogens, and limited local context integration.
Co-production of knowledge framework in Arctic research (Yua, et.al; 2022)	This framework fosters equitable Arctic research by deliberately bringing together Indigenous Peoples' and scientific knowledge systems to co-create context-rich understanding and solutions.	It enhances cultural relevance and inclusivity by centering trust, respect, and shared authority throughout the research process.	It demands considerable time, resources, and careful negotiation of power dynamics, requiring respect for Indigenous sovereignty and allowance for communities to opt in or out of research initiatives.

Fig. S1. Scoping review flow diagram according to PRISMA guidelines.

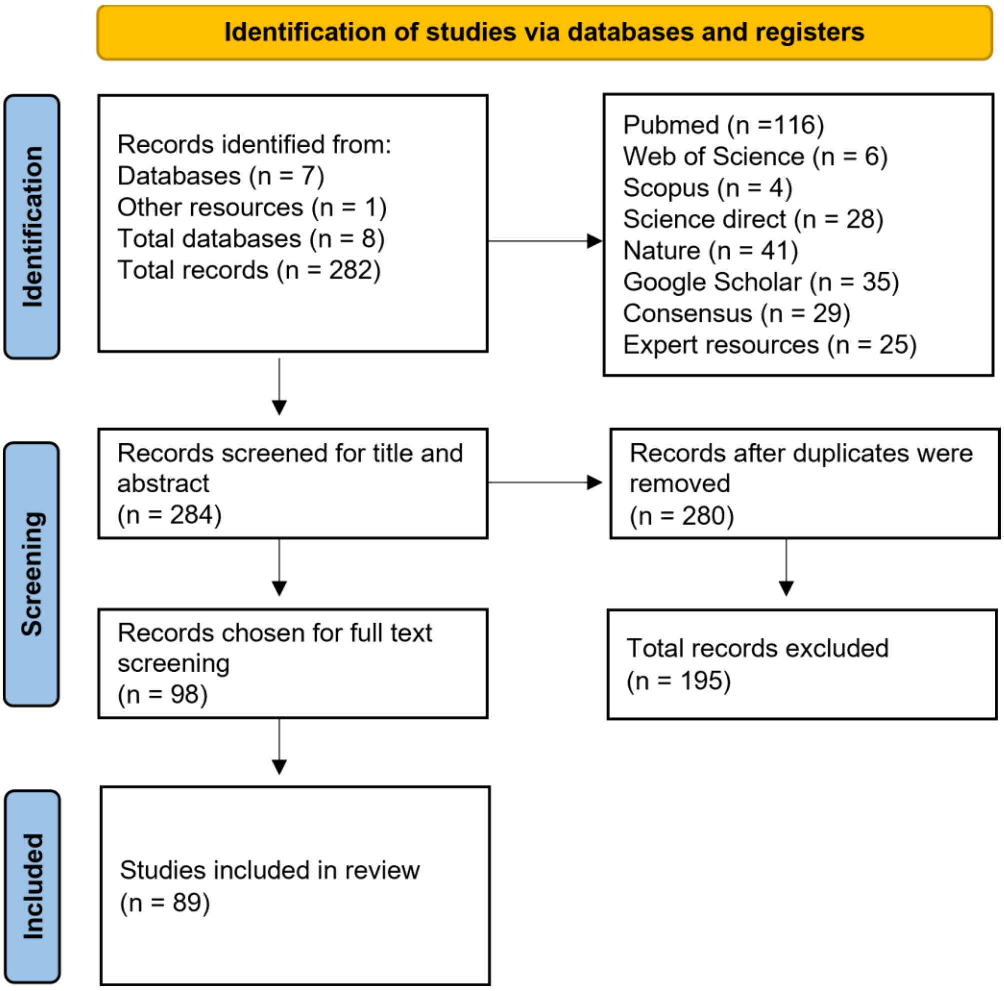

Supplement: Supplementary file 2 — Supplementary file2 (PDF 409 KB) [file 13280_2025_2255_MOESM2_ESM.pdf]
